# Discrimination of *Curculigo orchioides* Rhizoma and *Curculigo glabrescens* Rhizoma using stable isotope and mineral element analyses coupled with chemometrics

**DOI:** 10.1038/s41598-022-16851-7

**Published:** 2022-07-22

**Authors:** Yushi Liu, Yiping Guo, Sheng Gong, Minghao Yuan, Juanru Liu, Xiaohong Li, Zhong Wu, Li Guo

**Affiliations:** 1 State Key Laboratory of Southwestern Chinese Medicine Resources, Chengdu University of Traditional Chinese Medicine, Chengdu, China; 2 School of Pharmacy, Chengdu University of Traditional Chinese Medicine, Chengdu, China; 3 Sichuan GuoQiang Traditional Chinese Medicine Co., Ltd., Chengdu, China

**Keywords:** Drug regulation, Analytical chemistry, Medicinal chemistry

## Abstract

Correct species identification is crucial for ensuring the quality, safety, and efficacy of herbal medicine. Market research indicates that *Curculigo glabrescens* Rhizoma (CGR) was the major counterfeit of the medicine *Curculigo orchioides* Rhizoma (COR). To accurately discriminate COR and CGR remains a challenge, and it becomes even more difficult when the herbs have been heavily processed into a powder. In this work, combined with high performance liquid chromatography analysis, a novel component in CGR was discovered, and two stable isotopes (N%, C%, δ^15^N, δ^13^C) and nineteen mineral elements were determined along with multivariate statistical analysis to distinguish the authentic COR samples and counterfeit CGR samples. The results showed that there were significant differences between the mean value of N%, δ^15^N and δ^13^C according to the botanical origins. In addition, these two species can be differentiated by principal component analysis (PCA) and orthogonal partial least squares discriminant analysis (OPLS-DA) analysis. A linear discriminant analysis (LDA) model with a good classification rate (100%) and cross-validation rate (100%) was established. Hence, stable isotope and mineral element contents combined with chemometrics analysis could be considered as an effective and reliable method for discriminating the source species of COR and CGR.

## Introduction

*Curculigo orchioides* Rhizoma (COR) is the dried rhizome of *C. orchioides* Gaertn., which has a long history of using as an herbal medicine in China. Traditionally, COR is used to nourish the kidneys, strengthen the bones and muscles, and dispel cold and dampness^1,2^. COR and its preparations are widely used in clinical practice, and have a pharmacological activity such as preventing osteoporosis^3–5^, anti-tumor^6^, as well as anti-oxidant^2^, anti-depressive^7^, neuroprotective properties ^8^, and also improves learning ability^9^. *C. orchioides* Gaertn. is mostly grown in the wild and is mainly distributed in southwest provinces of China, along with limited resources and low yields. In order to protect this unique treasure and benefit local farming market, a cultivation and planting base of COR has been established in the city of Yibin, Sichuan Province. Local people often consider COR as a supplemental health product, such as in tea bags and alcoholic beverage^10^. The CO species have also been reported commercially used as health products in other countries like India^11,12^. Currently, the formulation of a commercially successful health care product that is in great demand contains COR^13,14^, and the wild resources of COR are gradually being depleted. The imbalance between the supply and demand in the trading market has led to an increase in counterfeits.

**Figure 1:**
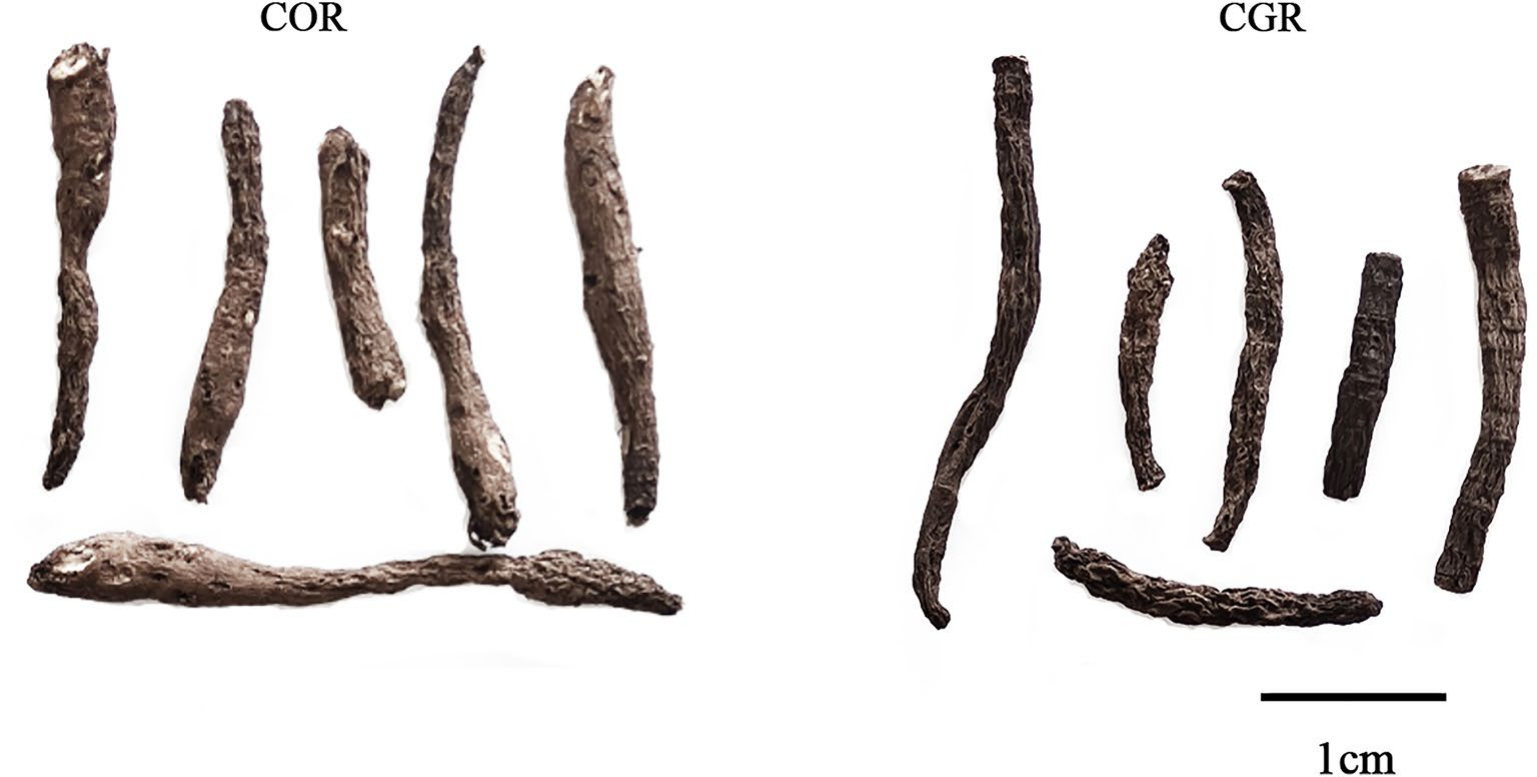
*Curculigo orchioides* Rhizoma (COR) (left) and *Curculigo glabrescens* Rhizoma (CGR) (right).

Correct species identification is crucial for the quality, safety, and efficacy of medicinal herbs^15^. Substitution and wrong identification often occur in clinical practice, when the medicinal herbs have similar morphological characteristics or names to be called^16^. In the market, *C. glabrescens* Rhizoma (CGR), which originated from Vietnam, has been aware as the major counterfeit of COR. Furthermore, CGR had yet been systematically reported, only a new compound and its free-radical scavenging activity, antidepressant activity had been reported^17,18^. There is no sufficient clinical evidence showing that CGR possesses pharmacological activity, the safety and effectiveness of CGR are still controversial. Therefore, the ability to accurately discriminate among the two source species is crucial for both market traders and consumers. COR and CGR come from different plants of the same species and genus, with close relationships, high genetic similarity, and similar morphological appearances^19^. To accurately discriminate those of two remains a challenge (Fig. 1), and it becomes even more difficult when the herbs have been heavily processed into a powder.

High performance liquid chromatography (HPLC) and curculigoside were applied to identify COR and CGR^20^, the preliminary research results showed that no significant difference was observed in the two species. The existing standards of Pharmacopoeia of the People's Republic of China (Ch.P, 2020 Edition) are determined by the methods of microscopic characteristics, thin layer chromatography characteristics, and chemical characteristics^21^, but these methods cannot provide the accurate distinction of COR and CGR. Thus, stable isotope ratios are considered to be an effective way to distinguish different geographical sources and species of herbs^22,23^. Generally speaking, each of the living organisms displays unique isotope compositions, especially in δ^13^C, δ^15^N values, owing to the differences between physical, chemical, and microbial isotope fractionation processes in different environments and species^24,25^. For example, the metabolic processes in C3 and C4 plants can cause differences in δ^13^C values^26^. It has been reported that inorganic elements contribute to the medicinal quality of traditional Chinese medicine plants^27,28^. Compared with the methods above, stable isotope and multielement analysis provided a high sensitivity and accuracy approach in the authentication of pharmaceuticals and herbal medicines. Stable isotope techniques and multielement analysis have been used to investigating the geographical origins and identifying the authenticity of food and traditional Chinese herbs, such as cereals^29^, honey^30^, Rhizoma *Coptidis*^27^, ginseng roots^26^, and wolfberry^31^.

To distinguish authentic COR samples and counterfeit CGR samples more accurately and conveniently, stable isotope ratios (N%, C%, δ^13^C, δ^15^N) and nineteen mineral element contents (Li, B, Na, Mg, Al, K, Ca, Ti, Mn, Fe, Co, Ni, Cu, Zn, Se, Sr, Mo, Cd, Ba) coupled with chemometrics were used. Different technologies were applied both individually and combined to establish better discriminatory models for COR and CGR. This study will be able to establish a stable identification method and provide reference and support for developing correct medication, market norms, and healthy utilization of COR.

## Materials and methods

### Chemicals and reagents

H_2_O_2_ and methanol of analytical grade were purchased from Chron Chemicals Co., Ltd (China), phosphoric acid and acetonitrile of HPLC grade were purchased from Fisher Chemical (American). Curculigoside (PS010133, purity > 98%) was purchased from Chengdu Push Bio-Technology Co., Ltd. HNO_3_ (65%) of MOS grade was purchased from Merck KGaA (Germany). Multielement standard solution (BWT30101-N-100) and Radix *Astragali* standard material (GBW10028) were obtained from Beijing Tanmo Quality Testing Technology Co., Ltd (China). USGS40, Wheat flour standard material was obtained from Elmental Microanalysis (Britain).

### Sample collection

Nineteen dried samples were collected from Sichuan Guoqiang Traditional Chinese Medicine Co., Ltd. (Chengdu, China). Ten samples were identified as COR by Sichuan Institute for Food and Drug Control (Chengdu, China), seven of COR from Sichuan (1–7), and three from Yunnan (8–10). Nine samples from Sichuan were authenticated as CGR (11–19) by Prof. Minru Jia (Chengdu University of Traditional Chinese Medicine). Voucher specimens were kept at the Chengdu University of Traditional Chinese Medicine (COR from 1 to 10: SCO0180812. COR from 11 to 19: SCG0191231). The study complies with the IUCN Policy Statement on Research Involving Species at Risk of Extinction and the Convention on the Trade in Endangered Species of Wild Fauna and Flora. The dry sample was milled to a fine powder using a pulverizing machine and passed through a 100-mesh sieve, then stored in a desiccator before other measurements. The study complies with local and national guidelines.

### HPLC analysis

Ch.P have made curculigoside as the only quality control marker. This is a nationwide standard, which stipulates the content of curculigoside (≥ 0.1%)^21^. Curculigoside reference solution was accurately prepared with methanol. COR and CGR powder (1.0 g each) were accurately weighed and added to 50 mL of methanol; the mixture was weighed and heated to reflux for 2 h. The mixture was cooled to room temperature and weighed again. Methanol was added to make up the lost weight, and the mixture was shaken and filtered through a filter paper. 20 mL aliquot of the filtrate was withdrawn and evaporated to dryness, and the residue was dissolved in methanol, transferred to a 10 mL volumetric flask, and methanol was added to the mark to obtain the sample solution. The solution was filtered through a 0.22 μm membrane filter before injection into the HPLC system.

The sample solutions of COR and CGR were analyzed by using Agilent 1260 HPLC (Agilent Technologies, CA, USA) system equipped with a Zorbax SB-C18 analytical column (4.6 mm × 250 mm, 5 μm) and a guard column. The temperature was set at 30 ℃, the injection volume was 10 μL, and the detection wavelength was set to 285 nm. Binary elution at a flow rate of 1.0 mL/min was employed using an aqueous phase of 0.1% phosphoric acid as solvent A and acetonitrile as solvent B; the isocratic elution procedure utilized A:B = 21:79; the detection time was 20 min^21^.

### Stable isotope ratios analysis

Elemental analysis with isotope ratio mass spectrometer (EA-IRMS, Vario EL III-Isoprime, Elementar, Germany) was used to determine the relative content of C and N elements and stable isotope ratios of the samples. 5.0 mg COR and CGR samples were weighed into a tin cap. In the analysis, the carbon contained in the samples was oxidized to pure CO_2_ by combustion (1150 °C), nitrogen was burned to form nitrogen oxides, and then reduced to pure N_2_ at a temperature of 810 °C. He was used as carrier gas and reference gas at a flow rate of 200 mL/min. The reference gases were CO_2_ and N_2_. USGS40 (δ^13^C_V-PDB_ = − 26.39 ± 0.04‰, δ^15^N_AIR_ = − 4.52 ± 0.06‰) and wheat flour standard material (δ^13^CV-PDB = − 27.21 ± 0.13‰, δ^15^NAIR = 2.85 ± 0.17‰) were chosen as the carbon and nitrogen isotope standards. The δ notation was used to report the isotopic difference between the sample and an international standard:

$$\updelta (\permil) = \left( {\frac{{R_{{{\text{sample}}}} }}{{R_{{{\text{standard}}}} }} - 1} \right) \times 1000$$

where *R* is the ratio of the heavy isotope to the light isotope, namely, ^13^C/^12^C and ^15^N/^14^N. The reference standards of δ^13^C, δ^15^N are Vienna Pee Dee Belemnite (V-PDB) and atmospheric, respectively^32^.

### ICP-MS determination

First, 0.5 g of sample was accurately weighed into Teflon digestion vessels, then 7 mL HNO_3_ and 1 mL H_2_O_2_ was added into the vessels. The digestion vessels were placed inside a microwave digestion instrument (ETHOS.SE, Milestone, Italy) and digestion was performed according to the following procedure: the samples were heated to 150 °C within 10 min and maintained for 2 min; the heat was increased to 180 °C within 3 min and maintained for 8 min and the samples were cooled. Finally, the digested liquid was transferred into a 50-mL volumetric flask and diluted with ultrapure water to the scale line for ICP-MS determination^33^. In addition, the sample was diluted 10 times for the determination of K, Ca. All materials were previously cleaned and kept in 10% HNO_3_ (v/v) and then rinsed three times with ultrapure water.

Nineteen mineral elements (Li, B, Na, Mg, Al, K, Ca, Ti, Mn, Fe, Co, Ni, Cu, Zn, Se, Sr, Mo, Cd, Ba) were determined inductively coupled plasma mass spectrometry (ICP-MS, iCAP RQ, Thermo Fisher, America). The operating conditions were as follows: RF power of 1550 W, a cooling gas flow rate of 14 L/min, an auxiliary gas flow rate of 0.8 L/min, a spray chamber temperature of 2.7 ℃, a peristaltic pump speed of 40 rpm, and a sampling depth of 5 mm. The internal standard elements Be, Sc, In and Bi were selected. Each sample was measured twice, and the relative standard deviation of the internal standard elements was required to be less than 5%. The LOD and LOQ were defined indicated as 3 σ and 10 σ, respectively. The standard material of Radix *Astragali* was used to evaluate the recovery and accuracy of the method. The rage recoveries of 19 elements ranging from 91.10 to 109.89%, indicating that this method can be used for sample determination (Table S1). The standard curve of elements was shown in Fig. S6.

### Statistical analysis

Statistical tests were carried out using SPSS 19.0 software, and the data were expressed as the mean ± SD. Statistical differences were identified by a *T*-test. A value of *P* < 0.05 was considered statistically significant. To reduce the dimension of data sets and describe all the variability of the system with fewer variables, principal component analysis (PCA), linear discriminant analysis (LDA), and orthogonal partial least squares discriminant analysis (OPLS-DA) were performed on the stable isotopes and multielement using the SIMCA-P software package. PCA is an unsupervised pattern recognition analysis that reduces the dimensionality of the data matrix. Original variables are transformed into principal components that are not related to each other through linear transformation^23^. OPLS-DA is well-suited for the classification of data that have multicollinear and noisy variables. In this study, the OPLS-DA procedure was used for cross validation when fitting to decide the significance of a component. The confidence level of parameters was set to 95%. According to the algorithm of OPLS-DA, the significant variables were selected^34^. LDA is a supervised model recognition algorithm that maximizes the variance between classes and minimizes the variance within classes by establishing new variables. The accuracy of LDA was verified by holdout cross-validation^23^.

## Results and discussions

### HPLC characteristic maker

**Figure 2:**
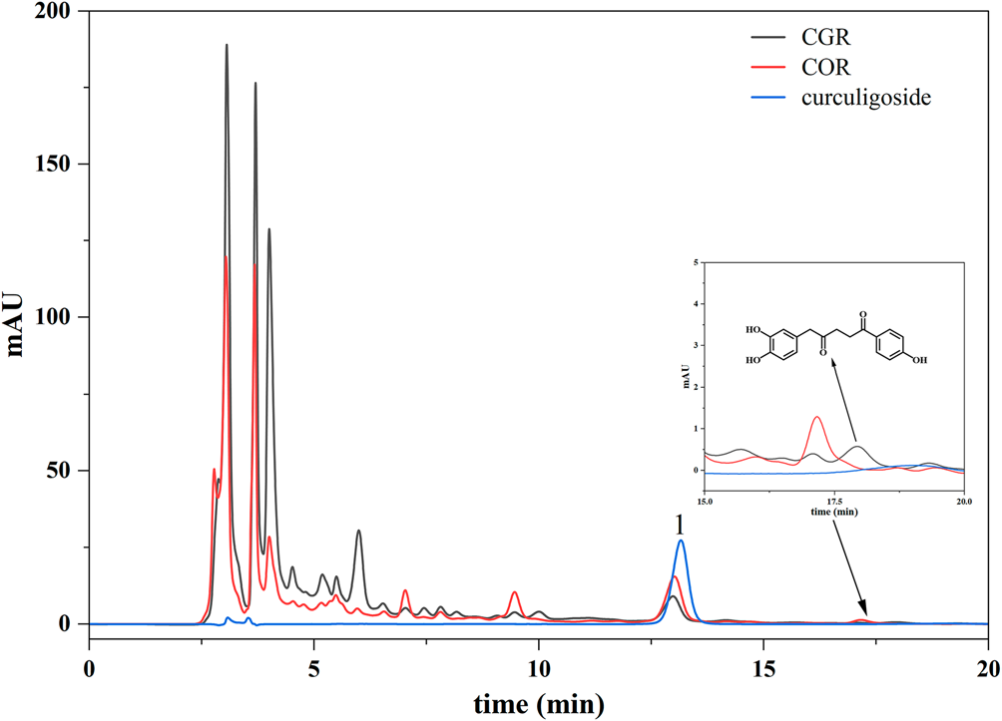
High-performance liquid chromatography (HPLC) chromatogram of curculigoside, COR and CGR samples. Peak 1: curculigoside.

Chemical features can be used to describe and evaluate medicinal materials as a whole. The HPLC method has good precision, sensitivity, and reproducibility, and can be used to quickly and specifically identify different herbs based on the overall chemical composition. The HPLC chromatograms of COR and CGR are illustrated in Fig. 2. The identities of the components were confirmed based on the retention time and ultraviolet spectra (285 nm) of the chemical markers. The main chemical components of COR and CGR were similar. As anticipated, the content of curculigoside (peak 1), the indicator component in COR, was not significantly from that in CGR. Interesting, CGR contains a unique compound that was detected in the HPLC chromatograms, but was not found in the profile of COR. Therefore, this unique compound was specifically separated and purified, and the structure was identified by modern spectroscopic techniques. It was a novel compound determined to be 5-(3′,4′-dihydroxyphenyl)-1-(4″-hydroxyphenyl) pentane-1,4-dione, 1D and 2D NMR spectra were available at Fig. S1–S5. However, the low content of this compound was not enough to accurately distinguish two plant sources.

### Variations in stable isotope ratios of COR and CGR

**Figure 3:**
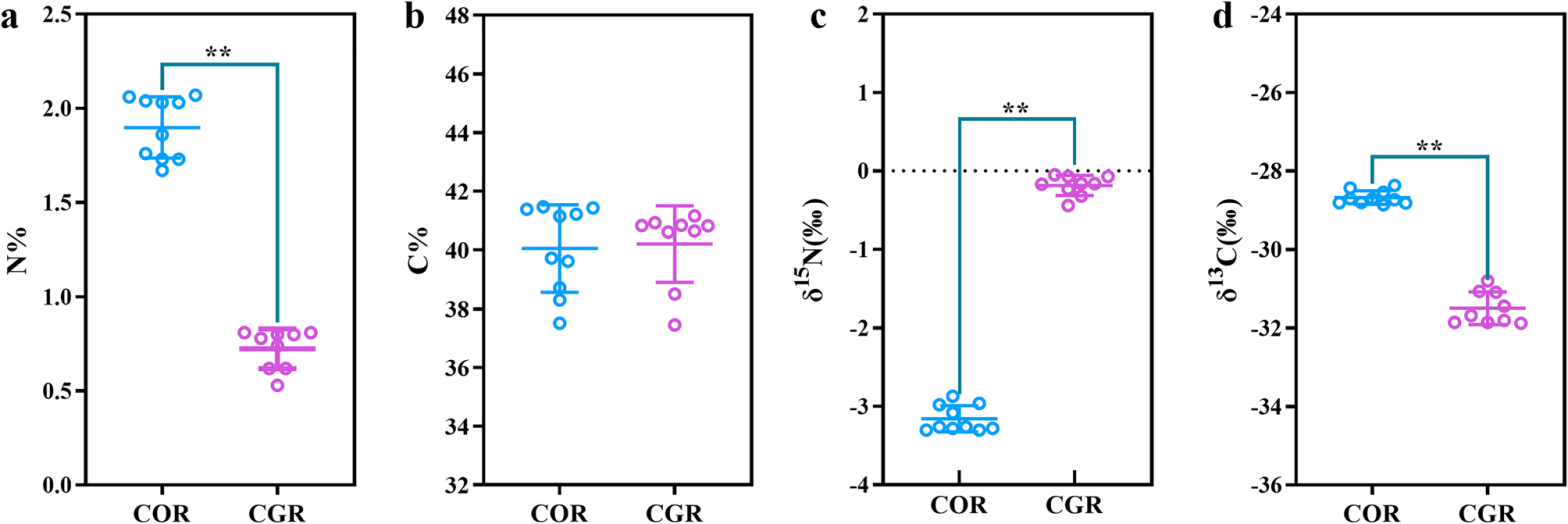
The relative content of N element (N%, **a**), C element (C%, **b**) and nitrogen isotope ratio (δ^15^N, **c**) and carbon isotope ratio (δ^13^C, **d**). Data were expressed as the mean ± SD. (**P < 0.01).

The variations in stable isotopic compositions between COR and CGR were shown in Fig. 3. The mean N% values of COR and CGR samples were 1.898% and 0.720%, the N% values in COR were significantly higher (Fig. 3a). The mean C% values of COR and CGR samples were 40.052% and 39.998%, respectively (Fig. 3b). The mean δ^15^N value of COR was − 3.157‰, which was significantly lower than the value of CGR, with the mean value of − 0.173‰ (Fig. 3c). The mean δ^13^C value of COR was − 28.678‰, which was significantly higher than the value of CGR, with the mean value of − 31.487‰ (Fig. 3d). There were significant differences in the mean value of N%, δ^15^N, and δ^13^C according to botanic origins (all *P* < 0.01 from *T*-test).

**Figure 4:**
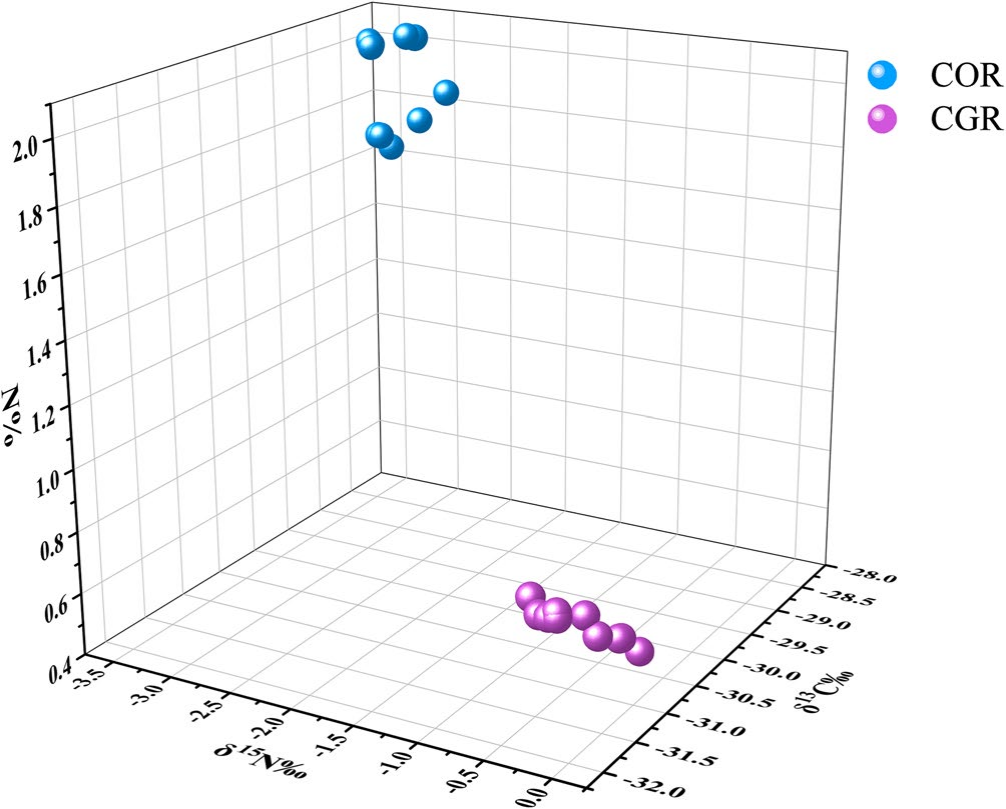
3D scatter plot of N%, δ^15^N and δ^13^C values in COR and CGR.

The 3D scatter plot of N%, δ^15^N, and δ^13^C values was presented in Fig. 4, and it exhibited the excellent ability to predict COR and CGR. On the whole, the COR had a high N% and δ^13^C value, and a low δ^15^N value, so they gathered at the top section in the 3D graph. However, the CGR, in contrast, mainly appeared at the bottom. The stable isotope ratio shows a good effect in distinguishing different sources of *Curculigo* Rhizoma.

### Mineral element analysis

The contents of mineral elements in COR and CGR samples were shown in Table 1. The results appeared significantly different among the two source species, except for B, Mg, K, Ca, Cu, Se, Ba. The K and Ca were the most abundant inorganic elements in COR and CGR. The Li, Al, Mn, Co, Ni, Zn and Cd contents were higher in COR than in CGR, while the concentrations of Na, Ti, Fe, Sr and Mo elements were present at a lower level in the COR samples.

**Table 1:** Average of mineral element concentrations (μg/g) of 10 COR and 9 CGR samples.

Elements	COR (n = 10)	CGR (n = 9)	*P*
Li	0.85 ± 0.19	–	0.003
B	7.60 ± 2.44	9.01 ± 1.26	0.053
Na	468.33 ± 114.57	717.20 ± 305.59	0.002
Mg	4852.03 ± 374.37	5059.72 ± 259.31	0.204
Al	389.68 ± 297.01	341.76 ± 25.63	0.000
K	14,582.97 ± 1947.35	10,401.14 ± 1739.31	0.799
Ca	12,836.15 ± 2524.55	17,847.83 ± 1393.4343	0.086
Ti	19.04 ± 1.65	34.35 ± 3.39	0.008
Mn	742.26 ± 265.38	288.31 ± 35.52	0.000
Fe	192.14 ± 209.94	327.22 ± 41.25	0.000
Co	3.39 ± 0.59	0.09 ± 0.07	0.002
Ni	9.51 ± 3.94	5.39 ± 2.03	0.011
Cu	22.51 ± 4.40	11.44 ± 2.40	0.055
Zn	271.42 ± 58.82	77.39 ± 14.03	0.000
Se	0.22 ± 0.03	0.03 ± 0.03	0.864
Sr	40.21 ± 6.94	149.95 ± 18.10	0.001
Mo	0.07 ± 0.05	0.70 ± 0.36	0.000
Cd	3.01 ± 0.60	–	0.000
Ba	220.54 ± 47.41	270.89 ± 27.93	0.052

Data were expressed as the mean ± SD. —means not checked out. The *P* < 0.05 reflects the statistical significance of the difference between groups.

### Principal component analysis of COR and CGR

**Figure 5:**
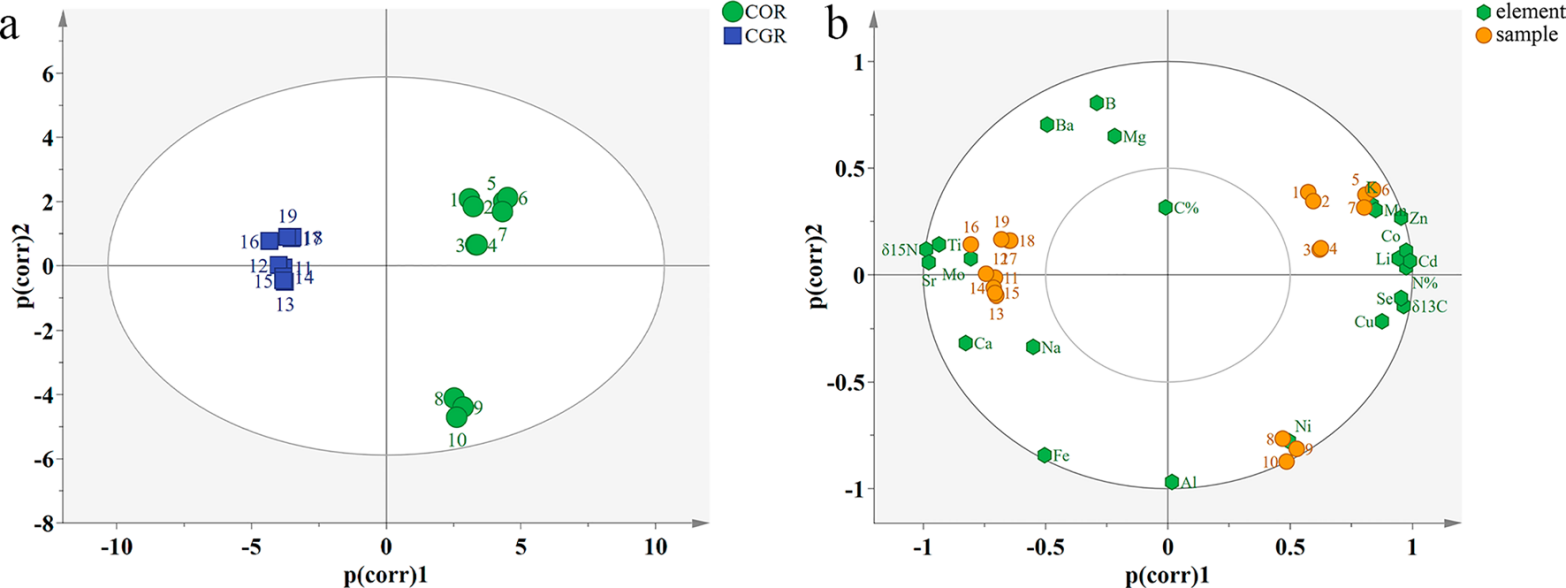
PCA classification result. Scatter plots of COR and CGR samples (**a**), PCA biplot for component PC1 and PC2 (**b**).

A multivariate evaluation is necessary to improve the overall accuracy of COR and CGR. Based on the chemical analysis of the stable isotope ratios combined with the concentrations of 19 mineral elements, the PCA analysis result was shown in Fig. 5a. The vectors and cumulative contribution of variance of the first three PCs (PC1-3) were shown in Table S3. A three-factor model (the first three PCs with eigenvalues > 1) can explain 88.0% of the total variability in the original data, which showed that the first three PCs can reflect most of the information in the samples. The PC1, PC2 and PC3 contributed 61.0%, 19.8% and 7.2% of the total variance, respectively. The result showed that 10 COR samples clustered together and 9 CGR samples clustered into another category. It was presented that COR and CGR samples can be well distinguished through PCA. Notably, three batches of COR from Yunnan tend to be distinguished from Sichuan.

The PCA biplot of PC1 and PC2 was presented in Fig. 5b. PC1 was mainly correlated with the intensity of N%, δ^13^C, Li, K, Mn, Co, Cu, Zn, Se, Cd and negatively correlated with δ^15^N, Sr, Mo signal^35,36^. The intensity of B, Al, Fe, Ni, Ba were important in PC2. COR samples (1–7) from Sichuan were mainly affected by the content of N% and elements Li, K, Mn, Zn, Co, Cd, while the COR from Yunnan (8–10) were isolated. PC1 had a better ability to discriminate COR samples. However, CGR samples (11–19) were clustered with δ^15^N, Ti, Sr, Mo. The classification of CGR was related to the content of these elements and can be distinguished by them. It was found that metabolic activities in plants had a greater impact on the content of δ^13^C than environmental factors^24,26^. Therefore, the difference between COR and CGR samples may be due to the different elements accumulated in plant metabolism.

### Identification of COR and CGR by OPLS-DA

**Figure 6:**
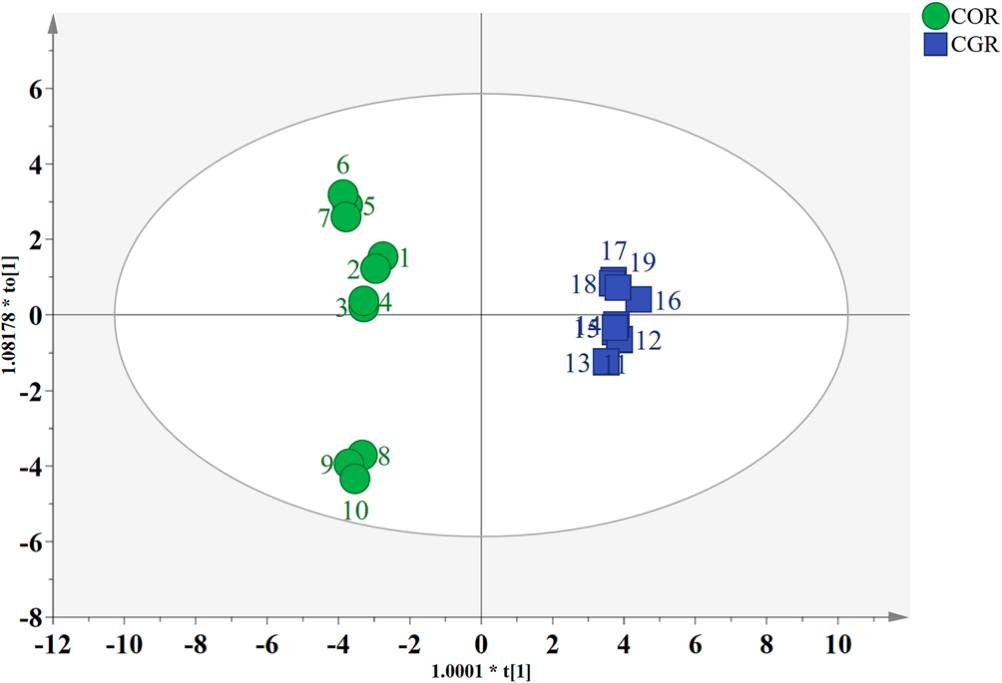
OPLS-DA classification result. Score plots showing the classification of authentic COR and counterfeit CGR samples.

To further utilize the potential discrimination capability of stable isotope and multielement analysis, OPLS-DA was used to process data related to COR samples and counterfeit CGR samples, and the result was shown in Fig. 6. The authentic COR samples and counterfeit CGR samples were significantly differentiated, indicating that stable isotope ratios and element contents combined with OPLS-DA analysis were an effective method to separate COR and CGR samples. The number of important components is determined by calculating the explained X variance (R^2^X), Y variance (R^2^Y), and the predictive ability of cross-validation (Q^2^) ^37^. The parameters for evaluating the OPLS-DA prediction models were as follows: R^2^X = 0.800, R^2^Y = 0.993, Q^2^ = 0.991. Generally, the model has the good fitting ability when these values are close to 1.0, the intersection point of R^2^ and Q^2^ with the Y-axis should be less than 0.3 and 0.05 respectively, and the difference between R^2^ and Q^2^ is less than 0.3^38,39^. Therefore, the results have shown that this OPLS-DA model was reliable. Moreover, VIP > 1 was considered as a good identification marker^27,34,40^, and OPLS-DA provided 13 effective potential markers (δ^15^N, Cd, Sr, δ^13^C, N%, Co, Se, Ti, Zn, Li, Cu, Mn, K) for determining the authenticity of COR samples and counterfeit CGR samples (Fig. S7). Notably, the three COR from Yunnan were also separated from COR samples from Sichuan based on their stable isotope ratios and element contents by the OPLS-DA model. The results indicated that stable isotope ratios combined with element contents might have the potential capability to predict the geographic origin of *Curculigo* Rhizoma. Based on these advantages, stable isotope ratios and element contents combined with OPLS-DA analysis is an excellent method of discriminating COR and CGR samples.

### Classification of Curculigo Rhizoma using LDA

To check the reliability of the classification model, LDA was performed using a cross-validation procedure to calculate the classification and probability of the COR and CGR samples^23,28^. The cross-validation result was displayed in Table 2. The LDA model gave a good classification rate (100%) and cross-validation rate (100%), COR and CGR were successfully identified. Thus, the predictive model performed well, LDA analysis combined with stable isotope and elements could be used to discriminate the two source species of *Curculigo* Rhizoma.

**Table 2:** Classification of COR and CGR samples based on discriminant analysis.

Predicted group membership			
	COR	CGR	Total
**Original**			
Count			
COR	10	0	10
CGR	0	9	9
Correct/%	100	100	100
**Cross-validated**			
Count			
COR	10	0	10
CGR	0	9	9
Correct/%	100	100	100

## Conclusion

In summary, COR and CGR could not be discriminated only by curculigoside. Stable isotope ratios (N%, C%, δ^15^N, δ^13^C) and nineteen mineral elements contents (Li, B, Na, Mg, Al, K, Ca, Ti, Mn, Fe, Co, Ni, Cu, Zn, Se, Sr, Mo, Cd, Ba) were determined to distinguish the herb species of COR and CGR. There were significant differences in the mean value of N%, δ^15^N, and δ^13^C according to botanical origins. Furthermore, stable isotope and multielement along with PCA analysis can be used to identify the authenticity of the two source species of *Curculigo* Rhizoma. A reliable OPLS-DA model was constructed to classify the authentic COR samples and counterfeit CGR samples. Meanwhile, effective potential markers for discriminating COR samples and counterfeit CGR samples were found. The LDA model with a good classification rate and cross-validation rate was established, which could be used to check the source species of *Curculigo* Rhizoma samples. In conclusion, stable isotope and mineral elements contents combined with multivariate statistical analysis could be an effective method for discriminating the source species of *Curculigo* Rhizoma, and provide a technical reference for the correct formulation of medication and establishing market norms. This method could potentially be applied to identify medicinal herb species.

## Supplementary Information

Supplementary Information.

## Data Availability

The data used in this study are available from the corresponding authors upon reasonable request.
