# Supplementary material for: Discrimination of Curculigo orchioides Rhizoma and Curculigo glabrescens Rhizoma using stable isotope and mineral element analyses coupled with chemometrics
Source: Sci Rep. 2022 Jul 22;12:12578. doi: 10.1038/s41598-022-16851-7 (PMC9307770; doi:10.1038/s41598-022-16851-7)
Supplement: Supplementary file 1 — Supplementary Information. [file 41598_2022_16851_MOESM1_ESM.pdf]

Discrimination of *Curculigo orchioides* Rhizoma and *Curculigo glabrescens* Rhizoma using stable isotope and mineral element analyses coupled with chemometrics

Yushi Liu[a, b, #], Yiping Guo[a, b, #], Sheng Gong[a, b], Minghao Yuan[a, b], Juanru Liu[a, b], Xiaohong Li[a], Zhong Wu[c] & Li Guo[a, b, ✉]

[a] State Key Laboratory of Southwestern Chinese Medicine Resources, Chengdu University of Traditional Chinese Medicine, Chengdu, China

[b] School of Pharmacy, Chengdu University of Traditional Chinese Medicine, Chengdu, China

[c] Sichuan GuoQiang Traditional Chinese Medicine Co., Ltd., Chengdu, China

✉ email: guoli@cdutcm.edu.cn

[#] Both authors contributed equally to this work

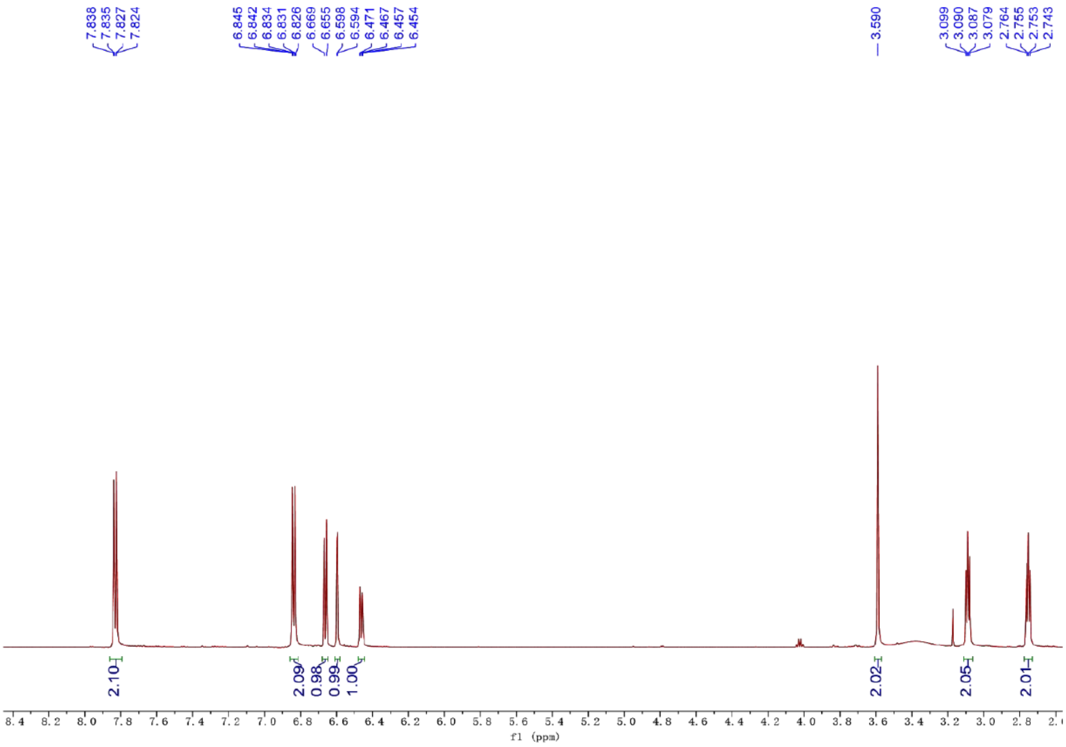

Figure S1. ^{1}H NMR of 5-(3',4'-dihydroxyphenyl)-1-(4''-hydroxyphenyl) pentane-1,4-dione.

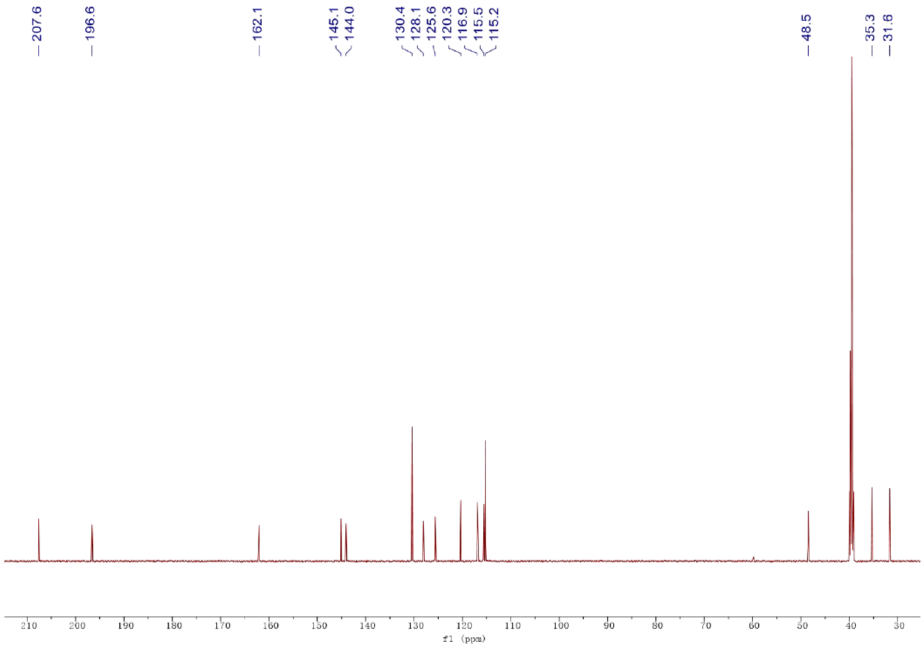

Figure S2. ^{13}C NMR of 5-(3',4'-dihydroxyphenyl)-1-(4''-hydroxyphenyl) pentane-1,4-dione.

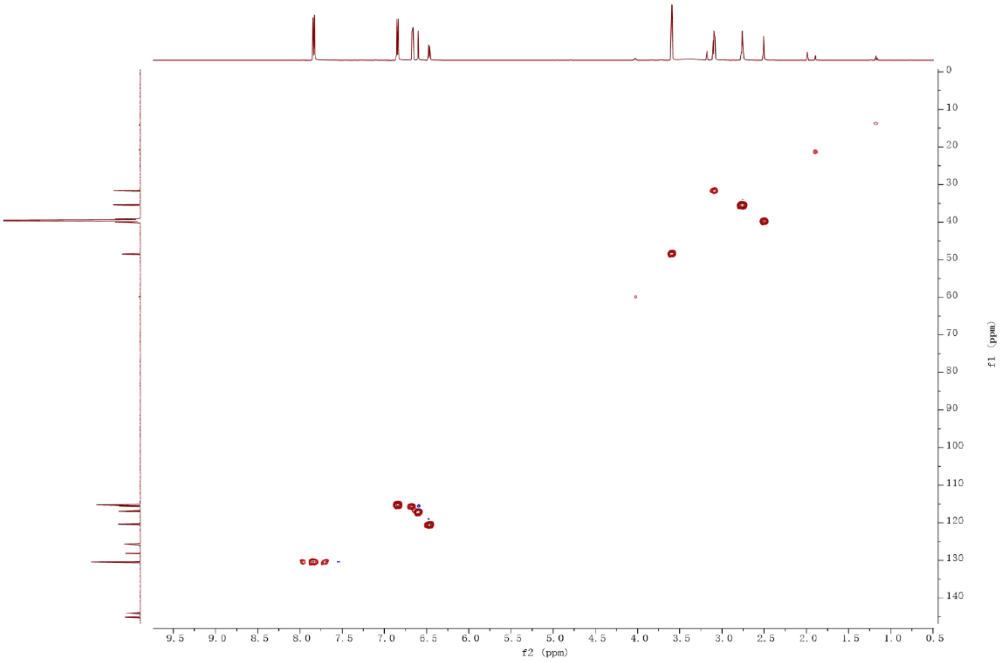

Figure S3. HSQC of 5-(3',4'-dihydroxyphenyl)-1-(4''-hydroxyphenyl) pentane-1,4-dione.

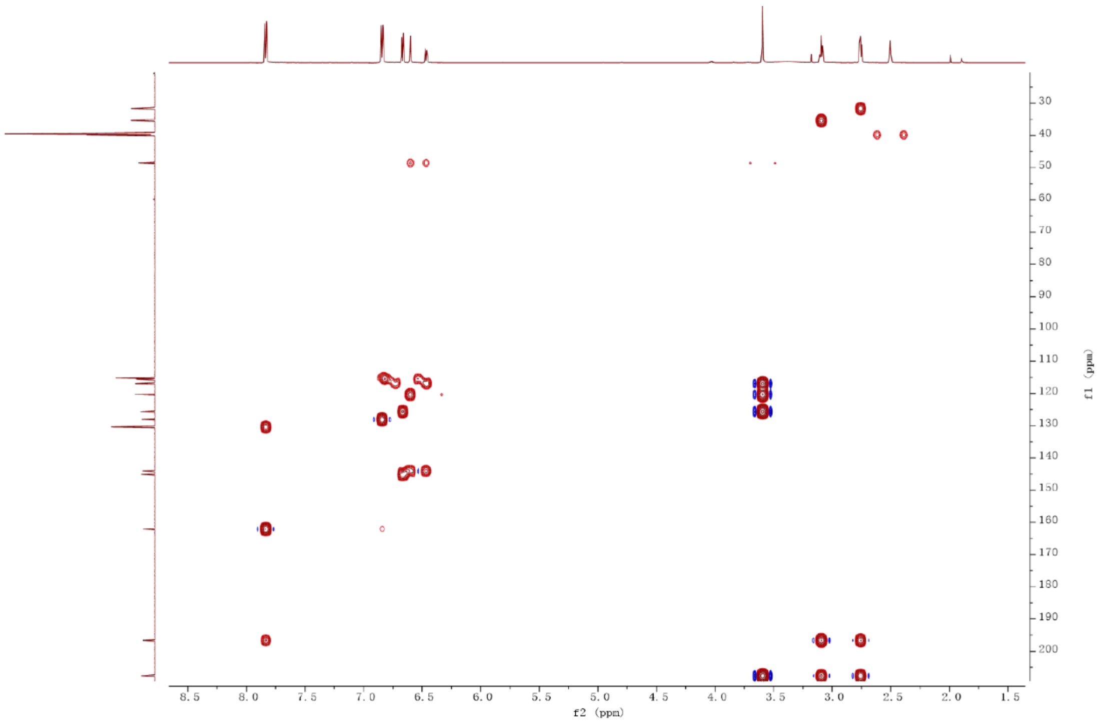

Figure S4. HMBC of 5-(3',4'-dihydroxyphenyl)-1-(4''-hydroxyphenyl) pentane-1,4-dione.

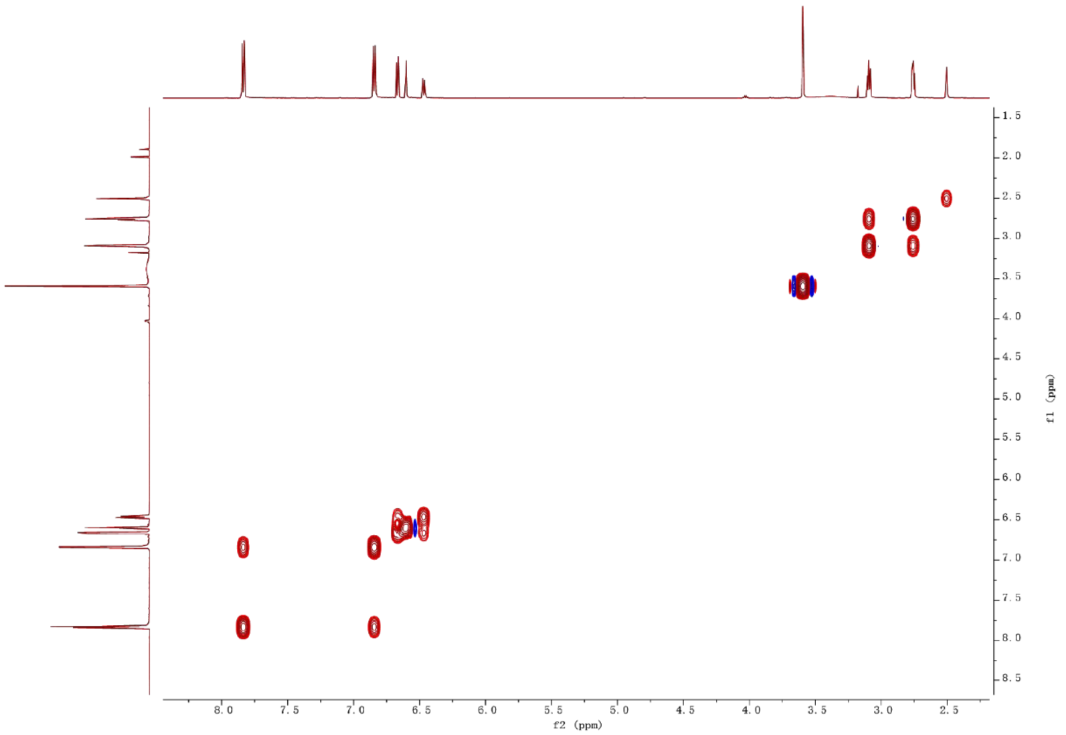

Figure S5. ^1H-^1H COSY of 5-(3',4'-dihydroxyphenyl)-1-(4''-hydroxyphenyl) pentane-1,4-dione.

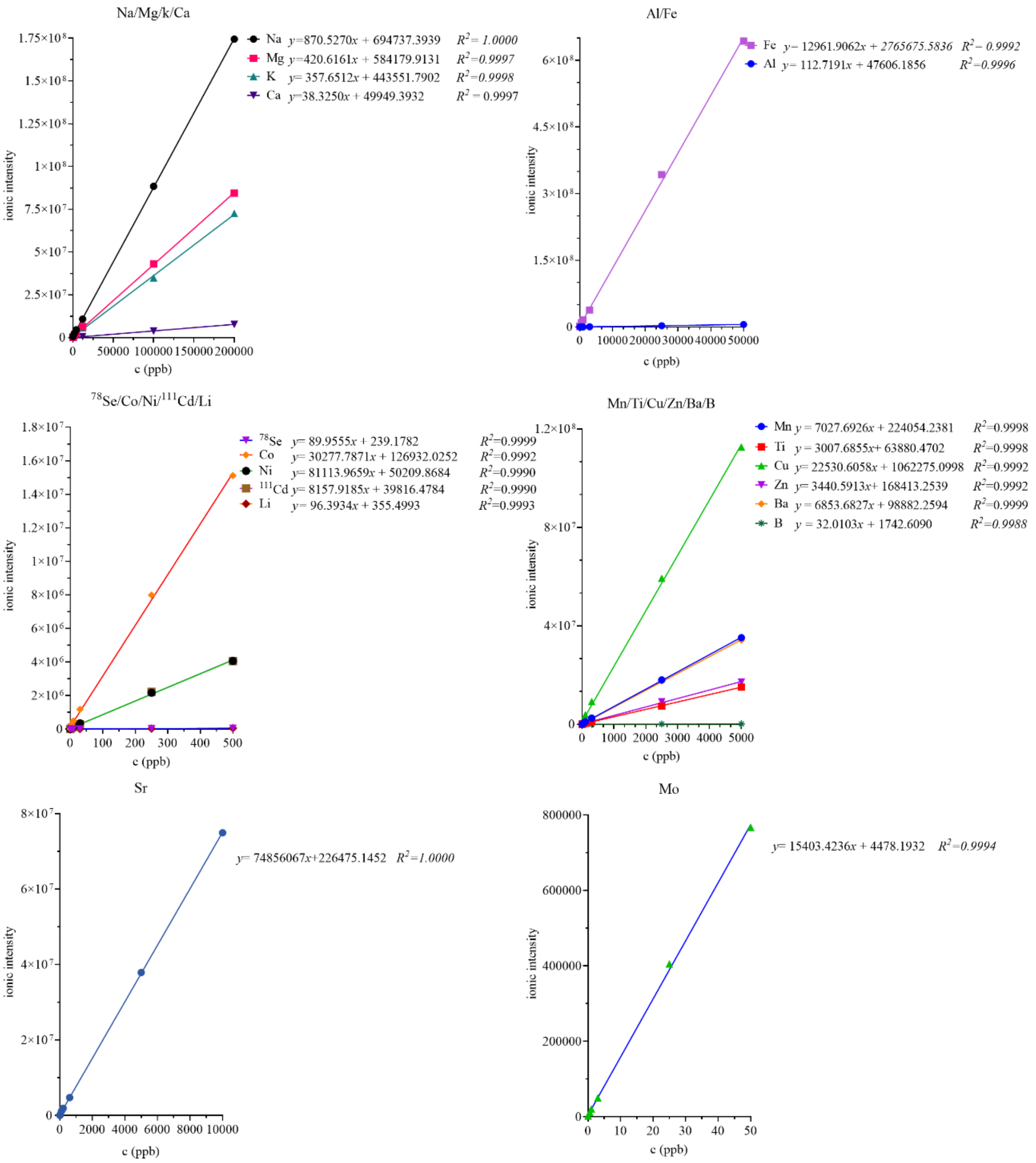

Figure S6. Standard curves of 19 mineral elements.

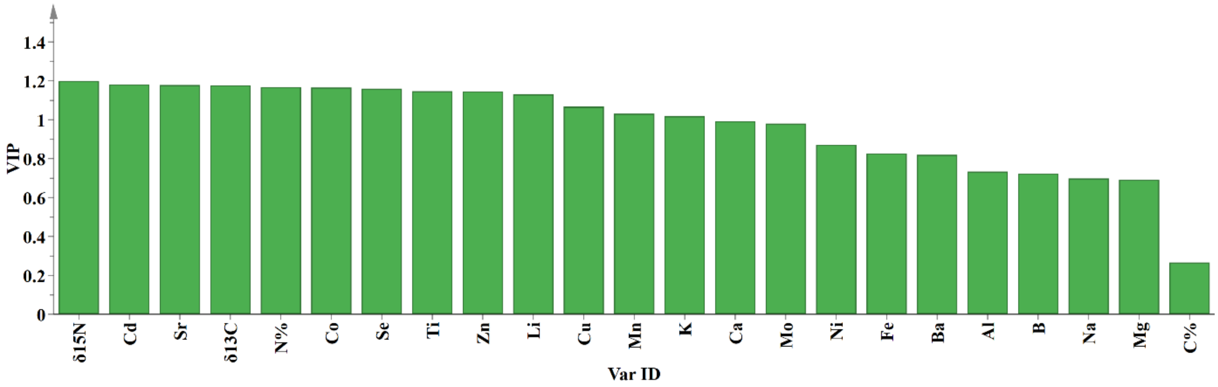

Figure S7. The VIP value of OPLS-DA model.

Table S1. Validation of the quality control of ICP-MS methods.

element	LOD (μg/g)	LOQ (μg/g)	Recovery/%
Li	0.013	0.043	96.51
B	0.044	0.147	97.32
Na	0.042	0.140	101.32
Mg	0.004	0.013	103.62
Al	0.036	0.120	96.98
K	0.071	0.237	101.28
Ca	0.023	0.077	103.53
Ti	0.006	0.020	96.02
Mn	0.012	0.040	109.89
Fe	0.038	0.127	93.53
Co	0.003	0.010	93.21
Ni	0.009	0.030	95.70
Cu	0.021	0.070	104.51
Zn	0.021	0.070	96.09
Se	0.002	0.007	98.82
Sr	0.007	0.023	91.10
Mo	0.006	0.020	92.08
Cd	0.003	0.010	108.51
Ba	0.039	0.130	99.66

Table S2. Elemental contents (μg/g) in the COR and CGR samples.

class	Li	B	Na	Mg	Al	K	Ca	Ti	Mn	Fe	Co	Ni	Cu	Zn	Se	Sr	Mo	Cd	Ba
COR-1	1.09	11.27	401.56	4863.02	194.96	14517.30	13156.50	21.03	648.74	43.45	4.13	7.88	15.80	310.15	0.22	48.51	0.16	2.83	269.02
COR-2	1.27	10.90	473.27	4868.71	197.35	14051.90	12737.20	20.36	640.34	41.36	4.16	7.68	15.03	298.33	0.22	46.69	0.11	2.96	251.41
COR-3	0.82	7.96	381.03	4647.90	213.01	13790.40	9784.14	16.06	595.58	80.05	3.57	6.00	20.87	257.37	0.19	30.63	0.06	2.66	247.27
COR-4	0.91	6.19	581.35	4615.98	214.00	13804.20	9904.50	16.41	578.52	74.67	3.42	5.89	20.12	257.69	0.25	36.24	0.07	2.50	256.81
COR-5	0.81	8.28	358.48	5214.12	207.65	17389.30	11662.70	20.30	1104.59	66.42	3.42	7.19	26.75	338.78	0.21	34.87	0.05	3.76	251.03
COR-6	0.76	8.81	360.91	5451.71	207.05	17346.30	11297.80	19.01	1118.73	71.16	3.73	7.57	26.94	329.89	0.24	33.26	0.04	3.86	232.72
COR-7	0.80	8.23	339.60	5382.09	203.54	16995.70	11350.90	19.94	1141.77	70.41	3.62	7.44	26.43	332.30	0.20	34.11	0.02	3.91	236.68
COR-8	0.73	4.95	557.70	4530.21	797.40	12711.50	16239.40	19.18	546.27	494.05	2.69	15.00	23.67	197.17	0.18	44.64	0.13	2.55	159.04
COR-9	0.64	5.24	581.41	4499.39	842.08	12620.60	15717.30	19.61	531.57	505.07	2.68	15.30	25.02	201.74	0.28	47.24	0.04	2.57	153.66
COR-10	0.69	4.12	648.06	4447.12	819.72	12602.30	16511.20	18.53	516.49	474.79	2.50	15.12	24.53	190.81	0.18	45.90	0.01	2.49	147.73
CGR-11	-	8.03	1066.47	4990.97	344.78	8367.39	16532.10	30.14	325.21	351.85	0.19	5.22	10.64	61.39	0.07	160.95	0.33	-	315.22
CGR-12	-	8.71	1042.02	4690.64	313.61	8106.61	16144.80	30.20	324.56	344.34	0.16	4.81	9.20	60.94	0.05	177.28	0.42	-	310.91
CGR-13	-	8.70	1030.77	4696.32	324.94	8116.71	15968.90	29.63	336.46	354.29	0.18	4.97	9.38	58.39	0.06	166.40	0.38	-	289.09
CGR-14	-	8.70	817.05	5228.22	381.74	11258.70	17336.40	36.26	250.76	387.96	0.09	8.04	14.22	78.04	0.03	129.46	1.28	-	237.51
CGR-15	-	6.93	800.60	5190.66	381.67	11465.90	18424.20	36.93	229.14	360.41	0.05	7.97	13.97	77.94	0.03	127.72	1.18	-	265.69
CGR-16	-	8.60	638.91	5500.21	316.26	10294.00	18415.60	34.82	284.16	305.95	0.10	1.07	7.44	95.99	-	165.70	1.02	-	248.56
CGR-17	-	9.83	358.48	5193.78	348.56	12121.60	19515.30	36.28	276.78	281.23	0.02	5.69	13.37	90.17	-	135.31	0.58	-	266.22
CGR-18	-	10.68	360.91	5072.04	339.14	11999.50	18758.00	36.68	283.96	285.37	0.02	5.52	12.90	87.13	-	140.23	0.52	-	253.28
CGR-19	-	10.90	339.60	4974.64	325.12	11879.90	19535.10	38.22	283.80	273.63	-	5.19	11.86	86.55	-	146.52	0.61	-	251.58

- means not checked out.

Table S3. The vectors and cumulative contribution of variance of the first four principal components.

Items	Principal component		
	1	2	3
N%	0.260	0.018	0.012
C%	-0.003	0.147	0.058
$\delta^{15}N$	-0.264	0.056	-0.014
$\delta^{13}C$	0.257	-0.069	-0.150
Li	0.252	0.036	-0.097
B	-0.077	0.377	0.055
Na	-0.147	-0.156	-0.460
Mg	-0.059	0.305	0.423
Al	0.005	-0.455	0.146
K	0.222	0.153	0.336
Ca	-0.221	-0.150	0.284
Ti	-0.250	0.068	0.236
Mn	0.227	0.141	0.093
Fe	-0.135	-0.395	0.081
Co	0.261	0.052	-0.078
Ni	0.133	-0.363	0.155
Cu	0.234	-0.100	0.219
Zn	0.255	0.126	0.048
Se	0.255	-0.052	-0.137
Sr	-0.261	0.028	-0.078
Mo	-0.215	0.037	0.242
Cd	0.264	0.030	0.039
Ba	-0.132	0.331	-0.345
Variance (%)	61.0	19.8	7.2
Cumulative variance (%)	61.0	80.8	88.0
